# Assessing the Organizational Climate for Translational Research with a New Survey Tool

**DOI:** 10.1007/s11948-020-00234-0

**Published:** 2020-06-26

**Authors:** Arno Simons, Nico Riedel, Ulf Toelch, Barbara Hendriks, Stephanie Müller-Ohlraun, Lisa Liebenau, Jens Ambrasat, Ulrich Dirnagl, Martin Reinhart

**Affiliations:** 1 German Centre for Higher Education Research and Science Studies (DZHW), Schützenstraße 6A, 10117 Berlin, Germany; 2 QUEST Center for Transforming Biomedical Research, Berlin Institute of Health, Berlin, Germany; 3 Department of Social Sciences, Humboldt-Universität zu Berlin, Berlin, Germany; 4 Charité - Universitätsmedizin Berlin, Berlin, Germany

**Keywords:** Translational research, Translation, Survey tool, Organizational climate, Research integrity, Biomedicine, Medicine, Clinical practice, Responsible research, Public health, Science, Research practice

## Abstract

Promoting translational research as a means to overcoming chasms in the translation of knowledge through successive fields of research from basic science to public health impacts and back is a central challenge for research managers and policymakers. Organizational leaders need to assess baseline conditions, identify areas needing improvement, and to judge the impact of specific initiatives to sustain or improve translational research practices at their institutions. Currently, there is a lack of such an assessment tool addressing the specific context of translational biomedical research. To close this gap, we have developed a new survey for assessing the *organizational climate for translational research*. This self-assessment tool measures employees’ perceptions of translational research climate and underlying research practices in organizational environments and builds on the established Survey of Organizational Research Climate, assessing research integrity. Using this tool, we show that scientists at a large university hospital (Charité Berlin) perceive translation as a central and important component of their work. Importantly, local resources and direct support are main contributing factors for the practical implementation of translation into their own research practice. We identify and discuss potential leverage points for an improvement of research climate to foster successful translational research.

**Electronic supplementary material:**

The online version of this article (10.1007/s11948-020-00234-0) contains supplementary material, which is available to authorized users.

## Introduction

Promoting the translation of knowledge through successive fields of research from basic science to public health impacts and back has become a central challenge for research managers and policymakers. One answer to this challenge is to support *translational research*, i.e. research that is oriented toward overcoming existing translational gaps (Drolet and Lorenzi 2011).^1^ Examples include interpretations of basic science to human application and clinical trails necessary to evaluate the safety and efficacy of interventions. Translational research is a crucial aspect of responsible research. Being translational in one’s research means being oriented toward application in clinical contexts and the overall aim of improving human health while avoiding ‘research waste’ in the form of ignored, irrelevant or poorly designed or conducted research (Chalmers et al. 2014; Ioannidis et al. 2014). The activity of designing and conducting translational research is what we call *translational research practices*.

Several attempts have been made to model translation and translational research as a linear, bidirectional, or circular process (Drolet and Lorenzi 2011; Rubio et al. 2010; Woolf 2008; Westfall et al. 2007; Sung et al. 2003), and it has been suggested that framing the process one way or another will have practical implications, e.g. on ‘how we train the next generation of researchers’ (Rubio et al. 2014). It has also become clear that supporting translational research is more than a matter of disseminating evidence about best practices to researchers and practitioners and expecting instant improvement. Instead, organizational leaders tasked with improving translational research at their institutions understand that translational research practices are diverse, highly context specific, and dependent on organizational cultures in support of translation (Simons et al. 2020; Hendriks and Reinhart 2018; Blümel et al. 2015, 2016; Rubio et al. 2014; Burke and Gitlin 2012).

So far, no tool exists that would allow organizational leaders to assess baseline conditions, identify areas needing improvement, and to judge the impact of specific initiatives to sustain or improve translational research at their institutions. To close this gap, we have developed and a survey for assessing the organizational climate for translational research and conducted initial validation steps. Our results indicate that this Survey of Translational Research Climate (STRC), which still awaits proper validation, could be used as a self-assessment tool to assess employees’ perceptions of translational research climate and potentially underlying research practices and conditions in organizational environments. Aggregated responses within meaningful organizational units provide a measure of group-level perceptions of environmental conditions, i.e. the ‘organizational climate’, for translational research.

Organizational researchers define organizational climate as ‘the shared meaning organizational members attach to the events, policies, practices, and procedures they experience and the behaviors they see being rewarded, supported, and expected’ (Ehrhart et al. 2013). Organizational climate is conceptually distinct from organizational culture, the latter of which is a ‘deeper-level construct’ (Ehrhart et al. 2013). Organizational culture puts the focus on judgements and values, while organizational climate puts the focus on organizational members’ perceptions of their organizational environment (Ashkanasy et al. 2011). However, the two concepts are intimately connected: organizational climate ‘is both the manifestation of culture…and the data on which culture comes to be inferred and understood’ (Reichers and Schneider 1990).

In developing the STRC, we built on the Survey of Organizational Research Climate (SOURCE) that assesses the organizational climate with respect to research integrity (Martinson et al. 2013; Crain et al. 2013). The STRC follows the general structure of the SOURCE, a validated questionnaire, but rephrases the questions in terms of translational research climate. As in the original SOURCE, the STRC distinguishes between two levels of analysis: 1) the immediate research environment (individuals’ primary subunit, such as research group or institute), and 2) the organization as a whole.

Consequently, we define translational research climate as the product of individual and group perceptions about their organizational unit—whether institution, division, department, center, program, or work group—in terms of how it values, is committed to, and administers its programs in ways that encourage the translation of research results between basic research, clinical research, and practice. Conceptually, this is different from assessing the climate for research integrity. Both integrity and translationality are aspects of responsible research, but while the former concentrates on cases of questionable, irresponsible formally defined misconduct, the latter focuses on the positive orientation of research toward its contribution to improving public health while avoiding ‘research waste’.

This paper reports a first implementation of this newly developed instrument at one of Europe’s largest university hospitals, the Charité – Universitätsmedizin Berlin. Results are compared against results from the original SOURCE.

## Methods

### Measures

The full questionnaire can be found in supplemental File 1. Here we describe its component parts.

### Translational Research Climate

To assess the climate for translational research, we have developed a standardized questionnaire, modeled after the SOURCE, whose 18 items fall into two groups: a) six items assessing the organizational climate for translational research on the institutional level, b) twelve items assessing the organizational climate for translational research on the level of one’s immediate research environment. Each item asks about the respondent’s perception of a particular aspect of the translational research climate in the organization as a whole or in the respondent’s immediate research environment, respectively, e.g. ‘How committed are the senior administrators at your institution (e.g. deans, executive board) to supporting translational research?’. Respondents rated items on a 5 point scale: (1) not at all, (2) somewhat, (3) moderately, (4) very, and (5) completely. To avoid forcing respondents to rate items, we offered two more response options: (6) no basis for judging, and (7) Prefer not to Disclose. This rating scale is identical to that of the SOURCE, apart from the very last option (‘Prefer not to Disclose’), which we added.

The selection and formulation of items underwent a three-step process. First, we assessed which of the 32 SOURCE items that could be adopted to our case, e.g. by replacing ‘responsible research’ by ‘translational research’. We settled on 18 items. The remaining 14 SOURCE items could not be translated in this way and were dropped, e.g. items about research misconduct. Second, we discussed our list of items among our project group, which included both medical researchers, experts on translational medicine, and social scientists. Third, to see how our items are interpreted and received within the target group, we conducted a cognitive pretest with five respondents of different status (graduate students, post docs, professors), gender, and working at different ends of the bench to bedside continuum (researchers, clinicians, clinician scientists).

### Translational Research Practices

We further included a set of 15 items asking about the respondents’ self-reported translational research practices (section 2C of the survey) to be able to relate these to the perceived translational research climate. We constructed these items to reflect six dimensions of translational research practices—(1) education, (2) communication, (3) publication, (4) collaboration, (5) career path, (6) overall. We identified these dimensions using qualitative data (literature review and interviews with 78 researchers, clinicians, and clinician scientists) from a previous research project on translation (Blümel et al. 2015, 2016). The items were discussed and tested, together with the translational research climate items, in our project group and in our pretest.

### Research Integrity Climate

We also administered the complete set of research integrity climate items of the original SOURCE—11 items for assessing institutional climate and 21 items for assessing the climate of the immediate research environment. This allowed us to investigate whether constructs in our translational research climate survey (STRC) were distinct from established constructs of research integrity climate (SOURCE).

### Other Measures

“Results” section of the survey included questions about the professional status at the institution, primary departmental affiliation, enrollment in a doctoral program, number of years working in research, whether one’s research is preclinical, clinical, or both, professional areas of interest, size of the immediate research environment, gender, and year of birth.

At the end of each survey (SOURCE and STRC), we included a final free text question to allow the respondent to make further comments, e.g. ‘Are there any other things about your experience with translational research practices at your institution that you would like to tell and about which we have not already asked?’

### Data Collection

Our study was approved by the Charité Ethics Committee (ethics vote number EA1/184/17) as well as the Data Protection Office and the Staff Council and was conducted as an anonymous web-based survey. For the use of the SOURCE, a license agreement was signed. The Charité administration helped us to identify all researchers and doctoral students working at the institution, 7264 individuals in total. We generated an equal number of electronical tokens and provided these to the study center’s administration, the latter of which created a linking table, assigning tokens to email addresses, and sent the researchers invitation emails with a token-unique-hyperlink to the online survey, followed by a reminder 2 weeks later. The emails were sent in the name of the Dean of the Charité. As an incentive to participate, per participant 2€ were donated to one of two preselected non-governmental organizations (NGOs) working in the field of health care, from which participants could choose after having finished the questionnaire. This study was preregistered on OSF (https://osf.io/qak8e/). The survey was online for a period of 4 weeks during February and March 2018. Of all 7264 invitees, 1095 opened the survey for at least one second, 969 answered at least the first question, 602 completed at least “Introduction” section (the SOURCE), 533 completed at least section 2B (the STRC), 523 completed at least section 2C (self-reported translational research practices). 521 invitees completed the whole questionnaire including all status and demographic items, resulting in a response rate of 7%. Informed consent was obtained from all individual participants included in the study.

A non-response analysis conducted after completion of the survey and sent to all invitees returned that a number of participants were interrupted during filling the survey but were not aware that they could continue later on or simply forgot to do so. Others were discouraged by the length of the questionnaire.

To guarantee the anonymity of the respondents, none of the authors had access to the study center’s linking table, assigning tokens to email addresses, and the study center, in turn, had no access to the raw data, linking tokens to individual responses. Three of the authors (AS, BH, MR) deleted the token column and further pseudonymized responses by aggregating birth years into 5-year intervals and omitting the answers of the free text questions. The resulting dataset and the corresponding codebook are available from the OSF database at 10.17605/OSF.IO/QAK8E.

### Statistical Analysis

**Fig. 1:**
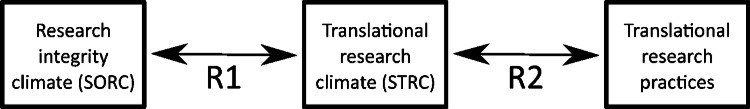
Relations between STRC and SOURCE. In the analysis of the STRC we focus on examining the overlap of STRC questions and the SOURCE questions (R1) as well as the relationship between STRC and translational research practice questions (R2)

When analyzing our data, we investigated two types of relationships, R1 and R2, as illustrated in Fig. 1. First, we established relevant factors for the STRC. Subsequently, we examined the identified factors for uniqueness with regard to the SOURCE questionnaire (R1). Finally, we investigated the relationship between the STRC and the items on translational research practice (R2). For the statistical analysis we used the statistical programming language R 3.5.1 (R Core Team 2018) with the additional packages ‘psych’ version 1.8.12 (Revelle 2018) and ‘lavaan’ version 0.6-5 (Rosseel 2012).

### Exploratory and Confirmatory Factor Analysis

To determine factors that summarize the answers to the STRC on a reduced number of scales we performed an exploratory factor analysis (EFA) on the 18 items from the STRC part of the questionnaire. For this, we largely followed the methods used for the development of the SOURCE scales (Martinson et al. 2013), but made some methodological changes where more robust methods were available. The full analysis code, including a commented out version that is identical to the analysis in the original SOURCE study, can be found on OSF at 10.17605/OSF.IO/QAK8E.

In summary, we mapped the answers to a numerical scale ranging from 1 (‘Not at all’) to 5 (‘Completely’). We then determined the number of factors by Horn’s parallel analysis (Horn 1965). The factor analysis was conducted using weighted least squares estimation, which is better suited for ordinal data compared to maximum likelihood estimation (DiStefano and Morgan 2014) and a oblimin rotation, which does not force the factors to be orthogonal. Each item was assigned to the factor for which it had the highest loading. No further modification was done for the factors after the analysis. For this analysis, participants were excluded who did not complete the questionnaire or gave answers ‘No basis for judging’ or ‘Prefer not to disclose’ more than 50% of the time for the STRC part of the questionnaire, leaving the answers of 438 participants for the analysis (i.e. 83 participants with > 50% ‘No basis for judging’ or ‘Prefer not to disclose’ answers for the STRC part).

As we had only one-third of the participant responses available as were used for the original SOURCE validation (438 full responses compared to 1267 responses for SOURCE) (Martinson et al. 2013), we chose a slightly different approach for the confirmatory factor analysis. Instead of initially splitting the full dataset into two parts, we used the full dataset for the EFA for the initial estimate of the factors. Then the dataset was split into two random parts n = 100 times. Each time the EFA was repeated on one half of the data to assess the robustness of the factors under different partitioning of the data. A confirmatory factor analysis (CFA) using diagonal weighted least squares estimation was then performed on the second half of the data, fixing the factors as determined in the EFA on the first half of the data. The average goodness of fit parameters (χ^2^/*df*, CFI, RMSEA, SRMR) were calculated for the CFA models to assess the adequacy of the fit. Again, all 18 STRC items were used for the CFA.

Additionally, we analyzed the relationship between the STRC and the SOURCE by calculating the correlation coefficients between the newly established STRC scales and the original SOURCE scales.

### Regression Modeling

Next, we analyzed the relationship between the STRC and the translational research practice questions using a regression analysis.

The following six dimensions of self-reported translational research practices were constructed prior to the conduction of the survey: overall (questions 2C01, 2C02), education (2C03), communication (2C04, 2C09, 2C10), publication (2C05, 2C06, 2C07, 2C08), collaboration (2C11, 2C12), career path (2C13, 2C14).

For the STRC questions, we used the factors that we established in the factor analysis. For both the STRC factors and the practice dimensions, we calculated the average scores for each factor/dimension as the mean of the scores for each question belonging to the factor/dimension. The average score was only calculated if participants answered more than 50% of the questions belonging to the factor/dimension.

To assess the relationship between the STRC factor scores and the practice dimension scores, we fitted a multiple linear regression model for each of the practice dimension using the three STRC factor scores as predictive variables. As the ‘career path’ dimension consists of two ‘yes/no’ questions, we fit a logistic regression model in this case. For this we categorized the answers for this practice dimension as follows: ((a) at least one of the two questions answered ‘yes’ (b) none of the questions answered ‘yes’. To account for the many different models that are tested, resulting *p* values were corrected using the Benjamini–Hochberg–procedure (Benjamini and Hochberg 1995).

The intraclass correlation (ICC) with respect to the institutions centers is calculated for the STRC factors to test if there is a difference in the perception of translational research practices within the surveyed institution.

For easier comparison with the results obtained in a study on the relationship between research integrity climate and practice for the SOURCE survey (Crain et al. 2013), we additionally repeated their analysis methods on the STRC (supplemental Table S3).

## Results

### STRC Factors

To determine into which coherent categories the items of the STRC can be grouped, we performed a factor analysis. The parallel analysis estimated that four factors are needed for the factor analysis. However, the EFA using four factors yielded that no question loaded mainly on the fourth factor. Thus, we decided to use three factors for the EFA instead.

As can be seen in Table 1, whereas factor 1 (‘Immediate environment’) only loads questions concerning the immediate research environment, factor 2 (’Institution’) only loads questions on the institutional level. Factor 3 (’Lack of resources and pressure’) combines four questions that deal with the lack of resources and publishing pressure. For all questions of factor 3, a larger score is inversely coded compared to the other questions, i.e. higher values denote a more negative view of the research environment.

**Table 1:** Factors obtained with the exploratory factor analysis for the STRC questionnaire

ID	Question text	Main factor	Factor 1 loading	Factor 2 loading	Factor 3 loading
P2B09	How consistently do responsible individuals in your immediate research environment communicate high expectations for translational research?	1	0.87	− 0.05	0.09
P2B02	How consistently does the overall ‘climate’ in your immediate research environment reflect high values for the translation of research?	1	0.83	0.04	− 0.04
P2B01	How committed are people in your immediate research environment to maintaining high standards of translation in their research?	1	0.82	0.03	0
P2B06	How committed are advisors in your immediate research environment to talking with advisees about key principles of translational research?	1	0.79	0.05	0.04
P2B05	How committed are people in your immediate research environment to making their findings ‘translatable/useful for others’?	1	0.78	− 0.07	− 0.03
P2B04	How reasonable are your immediate research environment’s expectations with respect to making your research useful for safe and effective health measures?	1	0.67	− 0.05	− 0.08
P2B08	How effectively are junior researchers socialized in translational research practices?	1	0.63	0.12	0.03
P2B11	How valued is envisioning safe and effective health measures in proposing, performing, and reporting research in your immediate research environment?	1	0.61	0.05	− 0.17
P2A02	How consistently does the overall ‘climate’ at Charite reflect high values for the translation of research?	2	− 0.1	0.81	0.02
P2A05	How committed are the senior administrators at Charite (e.g. deans, executive board) to supporting translational research?	2	− 0.04	0.76	− 0.06
P2A06	How effectively do the senior administrators at Charite (e.g. deans, executive board) communicate high expectations for translational research?	2	0.07	0.65	− 0.01
P2A03	How effectively do the available educational opportunities at Charite teach about translational research practices (e.g. lectures, seminars, web-based courses)?	2	0.07	0.64	0.07
P2A01	How committed are researchers at Charite to maintaining high standards of translation in their research?	2	0.13	0.63	0.01
P2A04	How accessible are individuals with appropriate expertise that you could ask for advice if you had a question about the translation of your research?	2	0.18	0.55	− 0.04
P2B12	How true is it that pressure to obtain external funding has a negative effect on making your research useful for safe and effective health measures?	3	0.02	0.07	0.81
P2B10	How true is it that pressure to publish has a negative effect on making your research useful for safe and effective health measures?	3	− 0.03	0.02	0.77
P2B07	How difficult is it to conduct translational research because of insufficient access to material resources such as space, equipment, or technology?	3	0.02	− 0.2	0.57
P2B03	How difficult is it to conduct translational research because of insufficient access to human resources such as expertise in research design, administrative or technical staff within your immediate research environment?	3	− 0.13	− 0.18	0.47

Questions for each factor are sorted according to factor loadings

Repeating the EFA on random 50% subsets of responses yielded a stable estimate of factors. While the factors stayed the same as for the EFA on the full data in 94 of the 100 cases, only one question is shifted between factors in the remaining 6 cases. The CFA applied on the other halves of the responses yielded average goodness of fit parameters that are slightly worse than those obtained for the original SOURCE (χ^2^ = 364, df = 132, CFI = 0.98, RMSEA = 0.089, SRMR = 0.091).

The average scores for the factors denote mean scores of all items belonging to a factor. The mean is not weighted by the EFA factor loadings, analogously to the SOURCE questionnaire. The average scores for the STRC factors for our survey responses are reported in Table 2. High values of Cronbach’s α, especially for the first two factors, demonstrate a good internal consistency of the factors.

**Table 2:** Average scores for the STRC factors

	Immediate environment	Institution	Lack of resources and pressure
n	463	413	457
Mean	3.21	3.03	2.92
SD	0.83	0.74	0.95
Reliability (Cronbach’s α)	0.91	0.84	0.75

The first row denotes the number of participants that answered more than 50% of the questions (not counting answers ‘No basis for judging’ or ‘Prefer not to disclose’) for each factor. The internal consistency of the factors is measured by Cronbach’s α. Note that Lack of resources is inversely coded

SOURCE versus STRC (R1)

While the mean scores for the original SOURCE scales were smaller than in the SOURCE publications, the standard deviations were very similar and the reliability coefficients are only slightly smaller (Table 3) (Wells et al. 2014; Martinson et al. 2013). Additionally, a comparison with the results of Haven et al. (2019), which used the SOURCE survey in several research institution in Amsterdam, including to academic medical centers, yielded mean scores for the SOURCE scales that are very similar to our results. The only exception in this case is the scale ‘Integrity Inhibitors’, which has a clearly higher mean score in our survey.

**Table 3:** Average scores for the original SOURCE scales

	Integrity norms	Integrity socialization	Integrity inhibitors	Advisor-advisee relation	Departmental expectations	RCR resources	Regulatory quality
n	492	498	507	509	496	492	398
Mean	3.67	3.13	2.80	3.42	3.22	3.01	3.13
SD	0.81	0.86	0.78	0.96	0.95	0.80	0.80
Reliability (Cronbach’s α)	0.80	0.81	0.68	0.87	0.74	0.81	0.79

The first row denotes the number of participants that answered more than 50% of the questions (not counting answers ‘No basis for judging’ or ‘Prefer not to disclose’) for each SOURCE scale. RCR abbreviates ‘responsible conduct of research’

The correlation analysis between the STRC and SOURCE scales yielded significant correlations for all comparisons between STRC and SOURCE scales (*p* < 0.001, range 0.22–0.68, average = 0.39; see supplemental Table S1), which are slightly lower than the significant correlations observed between the SOURCE scales (*p* < 0.001, range 0.24–0.66, average = 0.43), which were already found in Martinson et al. (2013). For this analysis we reverse-coded the two scales ‘Lack of resources and pressure’ and ‘Integrity Inhibitors’ such that for all STRC and SOURCE scales a larger score corresponds to a better research climate. For each STRC scale, one or two SOURCE scales can be identified with the highest correlation: ‘Immediate environment’ correlates strongest with the SOURCE scales ‘Integrity Norms’ and ‘Integrity Socialization’, ‘Institution’ correlates strongest with ‘RCR Resources’, and ‘Lack of resources and pressure’ correlates strongest with ‘Integrity Inhibitors’.

### Relationships Between STRC and Translational Research Practices (R2)

To investigate how the STRC factors relate to the different dimensions of translational practice, we performed a multiple linear regression analysis per practice dimension with the STRC factors as predictors. Unlike in the analysis of the relation between climate and practice for the SOURCE survey (Crain et al. 2013), we do not include any information on the institutional subunits in our analysis as the STRC factor scores show no substantial intraclass correlation (ICC) for the different centers of the surveyed institution (‘Immediate environment’: ICC = −0.0060, 95% CI [− 0.021, 0.036], ‘Lack of resources and pressure’: ICC = 0.012, 95% CI [− 0.011, 0.074], ‘Institution’: ICC = 0.030, 95% CI [− 0.0028, 0.116]).

**Fig. 2:**
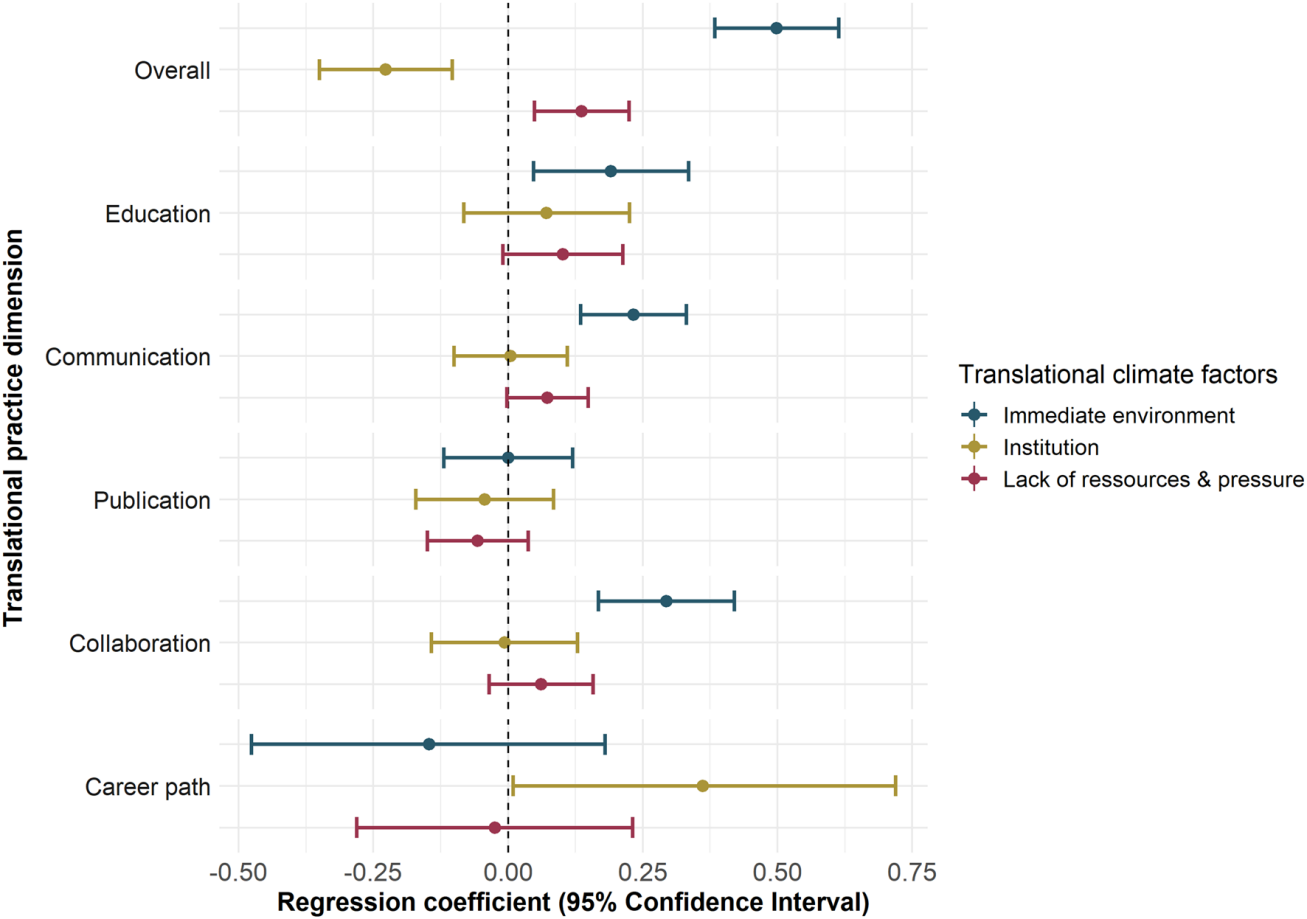
Regression coefficients for the STRC factors and their confidence intervals for all six regression models for the different practice dimensions. For the ‘Career path’ dimension, a logistic regression was used, such that this regression coefficient is not directly comparable with the other coefficients

As shown in Fig. 2, the connection is strongest and most consistent for the ‘Immediate environment’ factor, while the coefficient estimates were smaller for the ‘institution’ factor and the ‘Lack of resources and pressure’. The largest effect sizes (i.e. regression coefficients) were found for the variables ‘Immediate environment’ and ‘Overall’ (β = 0.50, *p* = 6.3 × 10^−15^), ‘Immediate environment’ and ‘Collaboration’ (β = 0.29, *p* = 3.8 × 10^−5^), ‘Immediate environment’ and ‘Communication’ (β = 0.23, *p* = 3.8 × 10^−5^) (supplemental Table S2).

The negative relation between the institutional climate and the overall practice (Fig. 2) is driven by respondents that give high scores for both the translational research climate in the immediate environment and the overall practice but low scores for the translational research climate in the institution.

The explained variance was calculated for each of the multiple linear regression models and ranges from R^2^ = 0.16 for the ‘Overall’ practice dimension to R^2^ = 0.005 for the ‘Publication’ dimension (supplemental Table S2).

In comparison with the SOURCE validation results (using the same methods, see supplemental Table S3) (Crain et al. 2013) our results give a similar magnitude of regression coefficients with less of the coefficients being significant—possibly due to the smaller participant number in our study.

## Discussion

We presented a new survey for assessing the organizational climate for translational research (STRC), which can be used as a self-assessment tool to assess employees’ perceptions of translational research practices and conditions in organizational environments. The Survey of Translational Research Climate (STRC) was modelled after the Survey of Organizational Research Climate (SOURCE), the latter of which assesses research integrity climate. While the STRC still needs better validation, the results from our study allow a positive outlook and suggest that the STRC could be a useful instrument with a distinct purpose, different from but complementing the SOURCE.

We developed the STRC in the context of a study with 521 respondents from the Charité, which also included the complete original SOURCE for the purpose of comparison. Three findings are crucial:

1. The STRC items discriminated into three factors, corresponding to three theoretical levels of analysis: (1) items concerning translational research climate in the immediate research environment, (2) items concerning translational research climate at the institutional level, and (3) items concerning publishing pressure and the lack of resources. STRC factors 1 and 2 showed good internal validity.
2. A correlation between certain scales of the STRC and the SOURCE suggests that even though research integrity and translation are concerned with quite different topics they might have some of the underlying problems in common. For example, the correlation between the STRC scale ‘Lack of resources and pressure’ and the SOURCE scale ‘Integrity Inhibitors’ might suggest that structural problems like the lack of resources are barriers for both research integrity and translational research.
3. We tested the relationship between the three STRC factors and six dimensions derived from additional items assessing the respondents’ self-reported translational research practices. That is, we investigated how perceived climate correlates with self-reported practice. Across dimensions, self-reported translational research practices were mainly correlated with the translational research climate in the immediate environment. Neither the institutional translational research climate nor the lack of resources showed strong correlations with translational research practices.

Several limitations apply. Even though the STRC is distinct from the SOURCE on most factors, our sample at one institution does not allow a generalization of the identified factors. Tests at additional institutions are necessary to confirm the exploratory results from our study. We ensured robustness of our results within our sample through cross-validation of our confirmatory factor analysis. In replications in other institutions this robustness test will reveal whether mapping between factors and questions will be equally stable as in our sample.

Our design tried to maximize response rate by sending reminders, nonetheless, we achieved a suboptimal return rate. An analysis of the demographics of the sample revealed a mixed picture on how representative our sample was for the institution given that we matched the gender ratio, but younger researchers like PhD students were over-represented in our sample. This may overestimate the effect that we find of the immediate research climate on translational research practices. Young researchers are less exposed to institutional structures like research committees and faculty structures, which manifests as a higher rate of ‘No basis for judging’ answers to the questions on the institutional climate for the youngest age group (supplemental Table S5). One issue that potentially caused the high abortion rate is the limited time researchers have to answer such questionnaires. In future installations, SOURCE questions should only be included as an additional voluntary set of questions to maximize return for the STRC.

Given our limitations, one result from our study is the positive relationship between immediate research environment and translational research practices. If that relationship is causal, which we did not investigate, interventions that aim at improving the translational research climate in the immediate research environment - such as investments in research infrastructure and education, incentives for interdisciplinary collaboration and better research quality, standards and guidelines, and more institutional support for bridging agents such as clinician scientists (Daye et al. 2015; Glasgow et al. 2004; Zerhouni 2003, 2007) - could be expected to have a positive effect on translational research practices (Strech et al. 2020). Note however that improvements in research environments and translational research practices in a single institution, such as a particular university hospital, cannot in themselves guarantee improvements in overall biomedical translation, when the latter is understood as a macro process or outcome, i.e. something that aggregates from distributed practices of a multitude of actors and institutions.

Assessing the perceptions of researchers, clinicians and clinician scientists about translational research practices and conditions at their institutions is novel and important, because it captures distinct aspects of responsible research, i.e. the positive orientation of researchers and their institutions toward improving public health while avoiding ‘research waste’ (Simons et al. 2020; Hendriks et al. 2019). We hope that the proposed STRC gets tested further and properly validated, which would be an important next step. If the STRC proves successful in this regard, it could become a standard tool for assessing, monitoring and improving translational research practices and policies, distinct from but complementary to the SOURCE.^2^

## Electronic supplementary material

Below is the link to the electronic supplementary material.

Supplementary material 1 (DOCX 43 kb)

## Footnotes

1 Translational research practices are different from non-research practices that help to improve translation, such as the implementation of clinical guidelines (Drolet and Lorenzi 2011).

2 In principal, the STRC and the SOURCE could be administered at the same time, i.e. handed out to the same people in a joint survey. In practice, however, we recommend separating the two assessments for practical reasons, mainly because shorter questionnaires likely result in higher response rates. While our findings confirm that the STRC and the SOURCE measure different concepts, respondents may get bored if they are asked to answer similarly phrased questions in two linked surveys.
