# Supplementary material for: Assessing the Organizational Climate for Translational Research with a New Survey Tool
Source: Sci Eng Ethics. 2020 Jun 26;26(6):2893–910. doi: 10.1007/s11948-020-00234-0 (PMC7755863; doi:10.1007/s11948-020-00234-0)
Supplement: Supplementary file 1 — Supplementary material 1 (DOCX 43 kb) [file 11948_2020_234_MOESM1_ESM.docx]

# Electronic Supplementary Material

**S1 Table. Correlation analysis of the STRC and SOURCE scales.** The correlation coefficients are given for each pairwise comparison of the STRC and SOURCE scales. All correlation coefficients are significant with p-values smaller 0.001. The scales are abbreviated as follows: STRC scales 1-3: Immediate environment, Institution, Lack of resources & pressure. SOURCE scales 1-7: Integrity Norms, Integrity Socialization, Integrity Inhibitors, Advisor-Advisee Relation, Departmental Expectations, RCR Resources, Regulatory Quality. For this analysis we reverse-coded the two scales ‘Lack of resources & pressure’ and ‘Integrity Inhibitors’ such that for all STRC and SOURCE scales a larger score corresponds to a better research climate. For better readability, we do not repeat the correlation coefficients but only give the values below the diagonal.

|  |  | STRC scales | SOURCE scales | | | | | | | | | |
| --- | --- | --- | --- | --- | --- | --- | --- | --- | --- | --- | --- | --- |
|  |  | 1 | 2 | 3 | 1 | 2 | 3 | 4 | 5 | 6 | 7 |  |
| STRC scales | 1 |  |  |  |  |  |  |  |  |  |  |  |
|  | 2 | 0.49 |  |  |  |  |  |  |  |  |  |  |
|  | 3 | 0.32 | 0.25 |  |  |  |  |  |  |  |  |  |
| SOURCE scales | 1 | 0.41 | 0.53 | 0.23 |  |  |  |  |  |  |  |  |
|  | 2 | 0.24 | 0.42 | 0.22 | 0.46 |  |  |  |  |  |  |  |
|  | 3 | 0.54 | 0.32 | 0.37 | 0.36 | 0.27 |  |  |  |  |  |  |
|  | 4 | 0.56 | 0.38 | 0.28 | 0.48 | 0.26 | 0.66 |  |  |  |  |  |
|  | 5 | 0.49 | 0.32 | 0.35 | 0.36 | 0.24 | 0.66 | 0.61 |  |  |  |  |
|  | 6 | 0.32 | 0.24 | 0.68 | 0.26 | 0.26 | 0.54 | 0.40 | 0.52 |  |  |  |
|  | 7 | 0.49 | 0.33 | 0.39 | 0.35 | 0.27 | 0.54 | 0.54 | 0.58 | 0.49 |  |  |

**S2 Table. Regression coefficients for the comparison of the three STRC factors and the predefined six practice dimensions.** One regression model is calculated per practice dimension with the three STRC factors as predictor. For each regression model the explained variance, F-statistic and corresponding degrees of freedom are given. For each regression coefficient the corresponding t-statistic and p-value are given. There are several significant coefficients (given p-values were corrected using the Benjamini Hochberg procedure), most of them involving the “Immediate environment” factor or the “Overall” practice dimension.

^§^As the ‘Career path’ dimension is comprised of ‘yes/no’ questions, a logistic regression model is used in this case. For this model the McFadden pseudo R^2^ and the χ^2^ statistic for the difference between null and residual deviance is used instead.

|  | Education | Communication | Publication | Collaboration | Career path | Overall |
| --- | --- | --- | --- | --- | --- | --- |
| Immediate Environment | 0.19^*^ (t=2.61, p=0.028) | 0.23^***^ (t=4.66, p=3.8x10^-5^) | -0.0002 (t=-0.003, p=0.99) | 0.29^***^ (t=4.58, p=3.8x10^-5^) | -0.15 (z=-0.87, p=0.53) | 0.50^***^ (t=8.52, p=6.3x10^-15^) |
| Institution | 0.07 (t=0.91, p=0.53) | -0.005 (t=-0.09, p=0.98) | -0.04 (t=-0.66, p=0.65) | -0.007 (t=-0.10, p=0.98) | 0.36 (z=2.00, p=0.12) | -0.23^**^ (t=-3.62, p=0.0015) |
| Lack of Resources & Pressure | 0.10 (t=1.79, p=0.15) | 0.07 (t=1.92, p=0.13) | -0.06 (t=-1.2, p=0.39) | 0.06 (t=1.24, p=0.39) | -0.02 (z=-0.19, p=0.98) | 0.14^**^ (t=3.06, p=0.0085) |
| Explained Variance (R^2^) | 0.034 | 0.070 | 0.0047 | 0.065 | 0.22^§^ | 0.16 |
| Test statistic | 4.53 (p=0.0039) | 9.71 (p=3.4x10^-6^) | 0.56 (p=0.65) | 9.02 (p=8.7x10^-6^) | 4.30^§^ (p=0.23) | 24.86 (p=9.8x10^-15^) |
| Degrees of freedom (d_1_, d_2_) | (3, 384) | (3, 389) | (3, 351) | (3, 389) | (3, 389) | (3, 389) |

**S3 Table. Regression coefficients of the comparison of the three STRC factors and the binary-coded six practice dimensions.** There are three significant coefficients (***p < 0.001 after Holm-Bonferroni correction), all involving the “Immediate environment” factor. Background: To allow for comparison with the results from a study on the relationship between research integrity climate and practice for the SOURCE survey (Crain et al. 2013), we repeated their validation analysis for the STRC dataset. For this we transformed the STRC practice dimension scores to a binary scale (analogously to their ‘always’/’not always’ categorization, we categorizing answers into high/low translational research practice, where all scores fall into the high category that are above the median score for this practice dimension). To assess the relationship between the STRC factor scores and the practice dimension scores we fitted a general linear model (GLM) with binomial error and logit for each combination of practice dimension as predictive variable and the binary version of the STRC factor score. To account for the many different models that are tested, resulting p-values were corrected using the Benjamini Hochberg procedure (Benjamini and Hochberg 1995). The magnitude of regression coefficients is similar to those for the SOURCE validation for the “Immediate Environment” factor and smaller for the other two factors. There were much less significant coefficients detected. This might be due to the smaller participant number in our study (521 participant responses used in the regression models compared to 1267 participants for the SOURCE validation). The binary coding of the practice scores was mainly used to compare the logistic regression coefficients to those presented for the SOURCE validation. When using the practice scores on their original 1-5 scale (see main text), we obtained the same three strongly significant regression coefficients plus several additional significant coefficients (note that the magnitude of the coefficients is interpreted differently in linear and logistic regression models).

|  | Education | Communication | Publication | Collaboration | Career path | Overall |
| --- | --- | --- | --- | --- | --- | --- |
| Immediate Environment | 0.32* (t=2.77, p=0.006) | 0.52^***^ (t=4.08, p=4.5x10^-5^) | 0.086 (t=0.70, p=0.48) | 0.54^***^ (t=4.49, p=7.0x10^-6^) | 0.004 (t=0.04, p=0.97) | 0.80^***^ (t=6.24, p=4.3x10^-10^) |
| Institution | 0.17 (t=1.28, p=0.20) | 0.32* (t=2.14, p=0.033) | 0.108 (t=0.76, p=0.45) | 0.22 (t=1.63, p=0.10) | 0.22 (t=1.48, p=0.14) | 0.02 (t=0.13, p=0.90) |
| Lack of Resources & Pressure | 0.04 (t=0.41, p=0.68) | 0.155 (t=1.44, p=0.15) | 0.036 (t=0.34, p=0.73) | -0.064 (t=-0.65 p=0.52) | -0.14 (t=-1.26, p=0.21) | -0.02 (t=-0.16, p=0.88) |

**S4 Table. Summary of the answers to the demographic questions.** We could not observe a strong difference between the study population with respect to gender (p=0.29, Fisher’s exact test) but we did observe a difference in the age distribution (p=1.0x10-7, Mann–Whitney *U* test), with especially more survey responses from very young persons (< 26 years), which might reflect a high participation rate for doctoral candidates.

| Category | Total | Percentage |
| --- | --- | --- |
| Age group |  |  |
| <26 | 40 | 7.7% |
| 26-30 | 92 | 17.6% |
| 31-35 | 87 | 16.7% |
| 36-40 | 73 | 14.0% |
| 41-45 | 45 | 8.6% |
| 46-50 | 43 | 8.2% |
| 51-55 | 26 | 5.0% |
| 56-60 | 17 | 3.3% |
| >60 | 8 | 1.5% |
| Prefer not to disclose | 91 | 17.4% |
| Gender |  |  |
| Man | 220 | 42.1% |
| Woman | 259 | 49.6% |
| Prefer not to disclose | 43 | 8.2% |
| Status |  |  |
| Stipend | 34 | 6.5% |
| Scientific Staff (‘WiMi’) | 263 | 50.4% |
| Junior research group leader | 24 | 4.8% |
| Junior professor | 1 | 0.2% |
| Professor | 58 | 11.1% |
| Other | 92 | 17.6% |
| Prefer not to disclose | 49 | 9.4% |
| Doctoral candidate |  |  |
| Yes | 205 | 39.3 |
| No | 290 | 55.6 |
| Prefer not to disclose | 27 | 5.2 |
| How many years in research |  |  |
| 1-3 years | 108 | 20.7% |
| 3-10 years | 194 | 37.2% |
| > 10 years | 206 | 39.5% |
| Prefer not to disclose | 14 | 2.7% |
| How would you describe your research? |  |  |
| Preclinical | 188 | 36.1% |
| Clinical | 135 | 25.9% |
| Both | 167 | 32.1% |
| Prefer not to disclose | 31 | 6.0% |

**S5 Table. Average percentage of ‘no basis for judging’ answers for the different parts of the questionnaire for all 521 respondents that completed the survey as well as the 40 respondents with age <26 years.** The questionnaire categories used are: STRC Institution – Questions 2A01 – 2A06, STRC immediate environment – 2B01 – 2B12, SOURCE Institution – Questions 1A03 – 1A11, SOURCE immediate environment – 1B03 – 1B19 and 1B22 - 1B23. For the full questionnaire as well as the question codes see <https://osf.io/qak8e/>.

|  | STRC institution | STRC immediate environment | SOURCE institution | SOURCE immediate environment |
| --- | --- | --- | --- | --- |
| All respondants | 26.2% | 17.0% | 17.8% | 7.7% |
| Age < 26 | 40.8% | 30.2% | 31.9% | 10.9% |

**S1 File. The complete STRC questionnaire, used to survey scientists at the Charité Berlin**

**The Survey of Translational Research Climate (STRC)**

*This survey is about* ***translational research*** *at your institution. Not everyone agrees on what the term ‘translational research’ means. When in doubt, please think of research practices that help to ‘translate’ findings in biomedical research into medical practice and meaningful health outcomes (this means, apply findings from biomedical research to medical practice, c.f. ‘from bench to bedside’). Please answer each of the following items with respect to the Charité as a whole (Part A), your immediate research environment (Part B), and your translational research practices (Part C).*

***Part A Charité as a whole***

| ***A01*** | How committed are researchers at Charité to maintaining high standards of translation in their research? | (1) Not at All  (2) Somewhat  (3) Moderately  (4) Very  (5) Completely  (9) No Basis for Judging  (10) Prefer not to disclose |
| --- | --- | --- |
| ***A02*** | How consistently does the overall "climate" at Charité reflect high values for the translation of research? | (1) Not at All  (2) Somewhat  (3) Moderately  (4) Very  (5) Completely  (9) No Basis for Judging  (10) Prefer not to disclose |
| ***A03*** | How effectively do the available educational opportunities at Charité teach about translational research practices (e.g., lectures, seminars, web-based courses)? | (1) Not at All  (2) Somewhat  (3) Moderately  (4) Very  (5) Completely  (9) No Basis for Judging  (10) Prefer not to disclose |
| ***A04*** | How accessible are individuals with appropriate expertise that you could ask for advice if you had a question about the translation of your research? | (1) Not at All  (2) Somewhat  (3) Moderately  (4) Very  (5) Completely  (9) No Basis for Judging  (10) Prefer not to disclose |
| ***A05*** | How committed are the senior administrators at Charité (e.g., deans, executive board) to supporting translational research? | (1) Not at All  (2) Somewhat  (3) Moderately  (4) Very  (5) Completely  (9) No Basis for Judging  (10) Prefer not to disclose |
| ***A06*** | How effectively do the senior administrators at Charité (e.g., deans, executive board) communicate high expectations for translational research? | (1) Not at All  (2) Somewhat  (3) Moderately  (4) Very  (5) Completely  (9) No Basis for Judging  (10) Prefer not to disclose |

***Part B Your immediate research environment***

| ***B01*** | How committed are people in your immediate research environment to maintaining high standards of translation in their research? | (1) Not at All  (2) Somewhat  (3) Moderately  (4) Very  (5) Completely  (9) No Basis for Judging  (10) Prefer not to disclose |
| --- | --- | --- |
| ***B02*** | How consistently does the overall "climate" in your immediate research environment reflect high values for the translation of research? | (1) Not at All  (2) Somewhat  (3) Moderately  (4) Very  (5) Completely  (9) No Basis for Judging  (10) Prefer not to disclose |
| ***B03*** | How difficult is it to conduct translational research because of insufficient access to human resources such as expertise in research design, administrative or technical staff within your immediate research environment? | (1) Not at All  (2) Somewhat  (3) Moderately  (4) Very  (5) Completely  (9) No Basis for Judging  (10) Prefer not to disclose |
| ***B04*** | How reasonable are your immediate research environment's expectations with respect to making your research useful for safe and effective health measures? | (1) Not at All  (2) Somewhat  (3) Moderately  (4) Very  (5) Completely  (9) No Basis for Judging  (10) Prefer not to disclose |
| ***B05*** | How committed are people in your immediate research environment to making their findings 'translatable/useful for others'? | (1) Not at All  (2) Somewhat  (3) Moderately  (4) Very  (5) Completely  (9) No Basis for Judging  (10) Prefer not to disclose |
| ***B06*** | How committed are advisors in your immediate research environment to talking with advisees about key principles of translational research? | (1) Not at All  (2) Somewhat  (3) Moderately  (4) Very  (5) Completely  (9) No Basis for Judging  (10) Prefer not to disclose |
| ***B07*** | How difficult is it to conduct translational research because of insufficient access to material resources such as space, equipment, or technology? | (1) Not at All  (2) Somewhat  (3) Moderately  (4) Very  (5) Completely  (9) No Basis for Judging  (10) Prefer not to disclose |
| ***B08*** | How effectively are junior researchers socialized in translational research practices? | (1) Not at All  (2) Somewhat  (3) Moderately  (4) Very  (5) Completely  (9) No Basis for Judging  (10) Prefer not to disclose |
| ***B09*** | How consistently do responsible individuals in your immediate research environment communicate high expectations for translational research? | (1) Not at All  (2) Somewhat  (3) Moderately  (4) Very  (5) Completely  (9) No Basis for Judging  (10) Prefer not to disclose |
| ***B10*** | How true is it that pressure to publish has a negative effect on making your research useful for safe and effective health measures? | (1) Not at All  (2) Somewhat  (3) Moderately  (4) Very  (5) Completely  (9) No Basis for Judging  (10) Prefer not to disclose |
| ***B11*** | How valued is envisioning safe and effective health measures in proposing, performing, and reporting research in your immediate research environment? | (1) Not at All  (2) Somewhat  (3) Moderately  (4) Very  (5) Completely  (9) No Basis for Judging  (10) Prefer not to disclose |
| ***B12*** | How true is it that pressure to obtain external funding has a negative effect on making your research useful for safe and effective health measures? | (1) Not at All  (2) Somewhat  (3) Moderately  (4) Very  (5) Completely  (9) No Basis for Judging  (10) Prefer not to disclose |

***Part C Your translational research practices***

| ***C01*** | Do you consider your own work translational? | (1) Not at All  (2) Somewhat  (3) Moderately  (4) Very  (5) Completely  (9) No Basis for Judging  (10) Prefer not to disclose |
| --- | --- | --- |
| ***C02*** | Overall, do you think translation is a relevant issue? | (1) Not at All  (2) Somewhat  (3) Moderately  (4) Very  (5) Completely  (9) No Basis for Judging  (10) Prefer not to disclose |
| ***C03*** | Since finishing your university education, how often have you acquired knowledge and skills beyond your main field of expertise (e.g., through continuous education, professional training, online courses, field visits) | (1) Never  (2) Rarely  (3) Sometimes  (4) Very Often  (5) Always  (10) Prefer not to disclose |
| ***C04*** | How often do you read journals/magazines from outside of your main field of expertise? | (1) Never  (2) Rarely  (3) Sometimes  (4) Very Often  (5) Always  (10) Prefer not to disclose |
| ***C05*** | How many of your publications are in journals/magazines from outside of your main field of expertise? | (1) None of my publications  (2) Less than half of my publications (3) About half of my publications (4) More than half of my publications (5) All my publications  (9) Does not apply  (10) Prefer not to disclose |
| ***C06*** | How many of your publications have you made freely accessible to everyone? | (1) None of my publications  (2) Less than half of my publications (3) About half of my publications (4) More than half of my publications (5) All my publications  (9) Does not apply  (10) Prefer not to disclose |
| ***C07*** | How often do you make your data freely accessible to everyone? | (1) Never  (2) Rarely  (3) Sometimes  (4) Very Often  (5) Always  (9) Does not apply  (10) Prefer not to disclose |
| ***C08*** | How often do you use data made freely accessible by others? | (1) Never  (2) Rarely  (3) Sometimes  (4) Very Often  (5) Always  (10) Prefer not to disclose |
| ***C09*** | How often do you provide expertise to people from other fields of expertise? | (1) Never  (2) Rarely  (3) Sometimes  (4) Very Often  (5) Always  (10) Prefer not to disclose |
| ***C10*** | How often do you seek expertise from people from other fields of expertise? | (1) Never  (2) Rarely  (3) Sometimes  (4) Very Often  (5) Always  (10) Prefer not to disclose |
| ***C11*** | How often do you collaborate with people from other fields of expertise? | (1) Never  (2) Rarely  (3) Sometimes  (4) Very Often  (5) Always  (10) Prefer not to disclose |
| ***C12*** | How often are you involved in interdisciplinary projects? | (1) Never  (2) Rarely  (3) Sometimes  (4) Very Often  (5) Always  (10) Prefer not to disclose |
| ***C13*** | During your career, have you ever switched fields? | yes/no  (10) Prefer not to disclose |
| ***C14*** | During your career, have you ever switched between lab and clinic? | yes/no  (10) Prefer not to disclose |

***Concluding question***

| ***D01*** | Are there any other things about your experience with translational research practices at the Charité that you would like to tell and about which we have not already asked? | Free Text |
| --- | --- | --- |

***Demographic information***

| ***E01*** | *In what year were you born?* | *YYYY*  *[] Prefer not to disclose* |
| --- | --- | --- |
| ***E02*** | *How do you identify your gender?* | *[] Man*  *[] Woman*  *[] Other*  *[] __________ (fill in the blank)*  *[] Prefer not to disclose* |
| ***E03*** | *What is your current status at Charité?* | *[] Stipend*  *[] Scientific Staff (“WiMi”)*  *[] Junior professor*  *[] Professor*  *[] Other (e.g. doctoral candidate without contract)*  *[] Prefer not to disclose* |
| ***E04*** | *What is your primary departmental affiliation?* | *Charite > Pulldown 15 ChariteCentren*  *[] Prefer not to disclose* |
| ***E05a*** | *Are you a doctoral candidate?* | *[] yes*  *[] no*  *[] Prefer not to disclose* |
| ***E05b*** | *If yes in III.1e: in which doctoral program?* | *[]* [Dr. med.](https://promotion.charite.de/promotion/promovend/dr_med_dr_med_dent/)  *[]* [Dr. med. dent.](https://promotion.charite.de/promotion/promovend/dr_med_dr_med_dent/)  *[]* [Dr. rer. medic.](https://promotion.charite.de/promotion/promovend/dr_rer_medic/)  *[]* [Dr. rer. cur.](https://promotion.charite.de/promotion/promovend/dr_rer_cur/)  *[]* [PhD](https://promotion.charite.de/promotion/promovend/phd_mdphd_und_dr_ph/)  *[]* [MD/PhD](https://promotion.charite.de/promotion/promovend/phd_mdphd_und_dr_ph/)  *[] Prefer not to disclose* |
| ***E06*** | *How many years are you working in research?* | *[] 0-3 years*  *[] 3-10 years*  *[] >10 years*  *[] Prefer not to disclose* |
| ***E07*** | *How would you describe your research?* | *[] preclinical*  *[] clinical*  *[] both*  *[] Prefer not to disclose* |
| ***E08*** | *Are you a clinician scientist?* | *[] yes*  *[] no*  *[] Prefer not to disclose* |
| ***E09*** | *What research area best describes your research?* | *Pulldown selected DFG areas*  *[] Prefer not to disclose* |
| ***E10*** | *Size of the immediate research environment you referred to in the questions above.* | *[] >10 people*  *[] 11-20 people*  *[] 21-50 people*  *[] 50-100 people*  *[] 100+ people*  *[] Prefer not to disclose* |
| ***E11*** | *Please choose which institution you would like to give your donation.* | *[] Deutsche Krebsstiftung*  *[] Open Science Foundation*  *[] at random* |
